# Leadership, staffing and quality of care in nursing homes

**DOI:** 10.1186/1472-6963-11-327

**Published:** 2011-11-28

**Authors:** Anders Kvale Havig, Anders Skogstad, Lars Erik Kjekshus, Tor Inge Romøren

**Affiliations:** 1 Norwegian social research (NOVA), Norway; 2 Centre for Care Research, Gjøvik University College, Norway; 3 Faculty of Psychology, University of Bergen, Norway; 4 Department of Health Economics and Health Management, University of Oslo, Norway

**Keywords:** nursing homes, leadership, staffing, quality of care

## Abstract

**Background:**

Leadership and staffing are recognised as important factors for quality of care. This study examines the effects of ward leaders' task- and relationship-oriented leadership styles, staffing levels, ratio of registered nurses and ratio of unlicensed staff on three independent measures of quality of care.

**Methods:**

A cross-sectional survey of forty nursing home wards throughout Norway was used to collect the data. Five sources of data were utilised: self-report questionnaires to 444 employees, interviews with and questionnaires to 13 nursing home directors and 40 ward managers, telephone interviews with 378 relatives and 900 hours of field observations. Separate multi-level analyses were conducted for quality of care assessed by relatives, staff and field observations respectively.

**Results:**

Task-oriented leadership style had a significant positive relationship with two of the three quality of care indexes. In contrast, relationship-oriented leadership style was not significantly related to any of the indexes. The lack of significant effect for relationship-oriented leadership style was due to a strong correlation between the two leadership styles (*r *= 0.78). Staffing levels and ratio of registered nurses were not significantly related to any of the quality of care indexes. The ratio of unlicensed staff, however, showed a significant negative relationship to quality as assessed by relatives and field observations, but not to quality as assessed by staff.

**Conclusions:**

Leaders in nursing homes should focus on active leadership and particularly task-oriented behaviour like structure, coordination, clarifying of staff roles and monitoring of operations to increase quality of care. Furthermore, nursing homes should minimize use of unlicensed staff and address factors related to high ratios of unlicensed staff, like low staff stability. The study indicates, however, that the relationship between staffing levels, ratio of registered nurses and quality of care is complex. Increasing staffing levels or the ratio of registered nurses alone is not likely sufficient for increasing quality of care.

## Background

The increasing number of older people in Norway combined with the lower ratio of persons of working age raises the quest for more efficient ways to organize and manage nursing homes. Thus, a central goal is - and will be - to identify which factors influence quality of care and how influential the different factors are. Several studies have recognized leadership as a key issue for quality of care in nursing homes [1-5]. There is limited knowledge of what kind of leadership behaviour that is related to quality of care, however [3,6-9]. Staffing has further been linked to quality of care in a number of studies [10-13]. In addition, staffing is emphasized in both the media and by the public as one of the most crucial elements for quality in nursing homes [14]

A weakness with some prior studies of leadership, staffing and quality of care in nursing homes is poor data quality. In particular the reliability of the staffing data [11,15-17] and the reliability and validity of the quality of care data [18-20] have been questioned. Furthermore, the majority of the previous studies in nursing homes have used secondary data sources to assess quality of care [6,9,13]. While secondary data sources have unquestionably advantages in relation to assess quality of care, there is a need for studies that base the quality assessment on primary data sources [13,21,22]. Therefore, in this study we collected rich data in 40 nursing home wards throughout Norway and performed a thorough analysis using both quantitative and qualitative methods. The definition of nursing home quality of care was based on *The national regulation for quality in nursing homes and home care *[23] and quality of care was assessed by three independent primary data sources: relatives, staff and field observations.

Quality in nursing homes is a multidimensional and elusive phenomenon and is complicated to define and assess [18,24-26]. In his classical work, Donabedian [27] suggested three approached to quality of care: structure, process and outcome. *Structure *referred to the general conditions that affect the ability to deliver care like staffing levels, staffing mix and characteristics of the nursing home, *process *referred to work processes, routines and procedures and *outcome *referred to the end result for the resident - do they receive good and adequate care? According to Donabedian [27], outcome measures were the ultimate validation of quality, but also the most complicated and time-consuming to measure. Donabedian's theoretical framework for understanding quality has been widely accepted among researchers [13,24,28,29].

Quality in nursing homes can also be divided in two dimensions: quality of care and quality of life [13,29,30]. Within this definition, *quality of care *encompasses clinical outcomes like the prevalence of pressure ulcer, falls or use of restrains, and focuses on the quality and safety of care. Contrary, *quality of life *encompasses residents' well-being and opportunities for choice, autonomy and meaningful social activities. Qualities of life comprise both an objective and a subjective dimension [29]. The *objective *dimension can be measured with "objective" indicators, while the *subjective *dimensions has focus on each individual perception of his or hers well-being.

To assess quality of care primary data sources and/or secondary data sources are used. *Primary data sources *are self-reported data from residents, relatives, care staff or field observations. Such data is generally time-consuming and expensive to acquire. *Secondary data sources *are typical national data sets of clinical assessments. The most common secondary data source is the MDS (Minimum Data Set). The indicators are normally calculated according to their presence or absence for an individual and then summed up for all individuals to create a facility level [19,31]. The MDS was not originally designed as a quality measurement instrument; however, researchers have increasingly derived quality indicators from the MDS data. Secondary data sources are the most common data sources of the two, particularly in the US [13].

In Norway, quality of care is regulated by *The national regulation for quality of care in nursing homes and home care *[23]. The regulation has been the starting point for indicators on quality of care in several studies [32-34]. According to the regulation, quality of care is a multidimensional phenomenon, consisting of a variety of aspects like medical care, general care, social activities, autonomy, interaction between staff and residents and privacy. Consequently, the regulation encompasses both a quality of care and a quality of life dimension.

### Leadership

Leadership has been studied using various approaches - traits, skills, styles and behaviour being the most common [35]. Regarding styles, a variety of different leadership styles have been identified and studied in the literature: Autocratic, democratic, directive, participative, task-oriented, relationship-oriented, transactional and transformational [36-40]. The present study focuses on two specific styles, namely task-oriented leadership and relationship-oriented leadership. Task-oriented style comprises the behaviours of *planning work activities *(what to do, how to do it, when to do it and who will do it), *clarifying roles and objectives *(communication of plans, policies, job responsibilities, role expectations, requirements and goals) and *monitoring operations and performance *(gathering information about the processes, progress, performance and individual contributions in the organisational unit). In contrast, relationship-oriented style constitutes the behaviours of *supporting *(consideration, acceptance and concern for the needs and feelings of subordinates), *developing *(building and developing subordinates' skills) and *recognising *(praising and showing appreciation toward subordinates for desired performance) [40]. A range of studies have investigated the effects of the two styles on a variety of outcomes - productivity indicators being among the most studied outcomes.

Studies on relationships between leadership styles and productivity are highly relevant for nursing homes, as quality of care is an essential indicator for the productivity level within the units [41]. In this research field there is a general agreement that both task-oriented and relationship-oriented leadership styles are systematically related to productivity. The effect of task-oriented leadership style on productivity has in these studies shown to be the strongest predictor of the two [35,37,40,42]. However, the effects of one style do not exclude the effects of the other. Rather, they complement each other. In line with this Yukl [40] states that: "The overall pattern of results suggests that effective leaders use a pattern of behaviour that is appropriate for the situation and reflects a high concern for task objectives and a high concern for relationship" (p. 130), while Northouse [43] states that: "The key to being an effective leader often rests on how the leader balances these two behaviours [task-oriented/relationship-oriented leadership style]. Together they form the core of the leadership process." (p. 44).

While several studies emphasize the importance of leadership for quality of care in nursing homes [1-5], few studies have investigated what kind of leadership influences the quality of care, and to what degree the two are related. In a systematic review of the relationship between nursing leadership and patient outcomes by Wong and Cummings [9], only seven studies met the inclusion criteria of "measuring leadership of a formal nurse leader" and "reporting a relationship between leadership and patient outcomes". Of these seven studies only one [1] investigated such relationships in nursing homes. In their literature review of leadership and outcomes in long-term care, Harvath et al. [6] concluded that "Despite the general consensus that leadership skills are important for nursing home nurses, we found very little evidence to support this claim." (p. 189). Eight studies met their inclusion criteria and linked leadership characteristics and outcome measures; however, only two of these studies included leadership behaviours. Anderson et al. [1], referred to in the literature study by both Wong and Cummings [9] and Harvath et al. [6], studied 164 nursing homes in Texas and found a significant negative relation between relationship-oriented leadership behaviours - as giving constructive feedback, helping staff resolve conflicts, generating trust and being approachable - and the prevalence of fractures and of complications of immobility. However, the studies showed no significant association between relationship-oriented leadership style and the outcome measures such as resident behaviour and restraint use. The task-oriented leadership behaviour of formalization, defined as specifying work procedures and rules and monitoring tasks, showed insignificant relationships with all of the four outcomes. McNeese-Smith [44], also included in Wong and Cummings' [9] literature review, interviewed 30 nurses working in a Los Angeles hospital and found that when staff nurses perceived their superiors to employ the typical relationship-oriented behaviours of support and conflict solving, the nurses systematically reported higher levels of productivity.

Three other studies, not included in the two literature reviews above, have shown interesting relationships between leadership behaviours and various quality outcomes. In a study of New York nursing homes, Hasemann [45] showed that authoritative leadership was systematically related to higher quality of care while nonauthoritative leadership was not. With authoritative leadership Hasemann [45] meant leaders who were delegating and telling, related to the followers both flexibly and decisively and made firm and impartial decisions. Nonauthoritarian leaders, by contrast, related to employees on a more personal level and were striving to please the employees and make them happy. Albinsson and Stang [46], in interviewing 32 experienced nursing home employees about how they thought the leader should function to achieve high quality of care, found that task-oriented behaviour characterized by well-defined leadership, goal formulation and care planning was emphasised as decisive in this regard. In a study at a relatively large Swedish hospital, Sellgren et al. [47] investigated the difference between staff and nurse managers in their preferences of leadership style for achieving high quality of care. They found that subordinates preferred leaders that took an active and clear leadership role and focused on production-orientated aspects of leadership rather than relationship-orientated aspects.

Because studies in nursing homes have been rather few and inconclusive, it is difficult to draw conclusions regarding which leadership style has the strongest effect on quality of care. In general, however, leadership studies has shown task-orientated leadership style to be the most influential of the two in relation to productivity - which in many cases overlap with quality of care. We therefore expected that both leadership styles will be systematically related to quality of care, but that the effect of task-oriented leadership will be the strongest.

### Staffing

The relationship between staffing - in the present study defined as *total staffing levels*, *ratio of registered nurses *and *ratio of unlicensed staff *- and quality of care in nursing homes has been debated by researchers for several years. The vast amount of studies in this field was recently illustrated in three literature reviews, identifying 87, 70 and 50 studies respectively [10,11,13]. Castle [11] identified 302 quality indicators among the 70 studies he examined. One hundred and twenty (40%) of these indicators were found to have a significant positive relationship with staffing levels, while 15 (5%) were found to have a significant negative relationship. Ninety eight out of the 302 quality indicators examined the effect of registered nurses and of these 51 indicators (52%) had a significant positive relationship with quality of care. Bostick et al. [10] concluded "that there is a proven positive association between higher total staffing levels and improved quality of care" (p. 366). Furthermore, Bostick [10] found support for an association between higher level of registered nurses and improved quality of care and a higher ratio of unlicensed staff and reduced level of quality of care. There are however, relatively few studies that have examined the effects of the ratio of unlicensed staff compared to studies which have examined the effect of staffing levels and the ratio of registered nurses, and the results of these are inconclusive [2,48-51]. In the most recent of the three literature reviews, Spilsbury et al. [13], found a tentative positive effect of increased total staffing levels and increased ratios of registered nurses on quality of care, however, they concluded that: "The existing evidence base does not enable any firm conclusions to be drawn when considering the relationship between nurse staffing and quality of care for residents in nursing homes." (p. 746). Ninety four percent of the studies examined were conducted in the US and the vast majority used secondary data sources to access quality of care.

Even though the majority of the studies in this research field have found that staffing levels and the proportion of registered nurses are significantly related to quality of care, a significant number of studies have not been able to link the two predictors and the specified criterion. For example, Rantz et al. [4] investigated 92 nursing homes in Missouri and did not find any effect for either staffing levels or staff mix. The study had reliable staffing data and a robust design, with data collected both quantitatively and qualitatively, the latter by two to four days of participating observations at each nursing home. Likewise, Winsløw & Borg [52] surveyed 7500 care workers in 36 municipalities in Denmark and found no association between increased staffing levels or level of professional training and quality of care provided by the individual care worker. Furthermore, Arling [2] and Berlowitz [53] did not find any significant relationship between staffing levels or staff mix and quality of care in their studies. In a Norwegian setting, three studies have investigated the relationship between total staffing levels and quality of care in nursing homes. Two of the studies found no significant relationship [33,54], while one of the studies found a significant positive effect of increased staffing levels on quality of care [32]. Harsvik et al. [54] also studied the effect of increased ratios of registered nurses on quality of care; however, they found no significant effect.

Based on the earlier studies it is still an open question to what degree staffing levels, the ratio of registered nurses and the ratio of unlicensed staff are systematically related to quality of care in nursing homes. The results of the studies are mixed and the reliability and validity of the data, particularly those studies using OSCAR data (Online Survey, Certification and Reporting), may be questioned [11,15-17]. Further, the studies are often inaccurate concerning whether they refer to the absolute level or the relative ratio of care staff. However, the majority of the studies indicate a positive effect on quality of care for higher staffing levels and higher ratios of registered nurses and a negative effect on quality of care for higher ratios of unlicensed staff.

### Aim

The purpose of the present study is twofold: 1) to assess the effect of ward leaders task- and relationship-oriented leadership styles on quality of care in nursing homes and 2) to assess the effect of staffing levels, ratio of registered nurses and ratio of unlicensed staff on quality of care in nursing homes.

## Method

### Research design

A cross-sectional design was used to collect the data required to test our research questions. Five different sources of data were utilised: self-report questionnaires distributed to 444 employees in nursing homes, interviews with and questionnaires to 13 nursing home directors and 40 ward managers, a telephone survey with 378 relatives and 900 hours of field observations in 40 wards.

### Sample

One to four wards in 21 nursing homes participated in the study, yielding a total of 40 wards. Nursing home ward was used as measurement unit due to the assumption that both leadership style and quality of care may vary significantly from one ward to another within one nursing home. The facilities were located in towns in eleven medium (6,000 - 20,000) and large-sized (> 20,000) municipalities in seven counties (Finnmark, Nord-Trøndelag, Hordaland, Hedmark, Oslo, Akershus and Aust-Agder) across Norway. The seven counties were selected to achieve geographical spread. Special care units for dementia were excluded, as such wards often have a different structure and relatively more staff than general wards. All nursing homes were public and nonprofit in nature, and owned and run by their local municipalities. The nursing homes ranged in size from 20 to 152 beds, with a mean of 63; the wards ranged in size from 7 to 34 beds, with a mean of 18. The number of staff (full-time equivalents) per ward ranged from 6 to 25, with a mean of 14. Staff was grouped according to number of working hours per week, but respondents were not categorised by the occupational categories of registered nurse, auxiliary nurse and unlicensed staff. Several of the participating wards had only two or three registered nurses, hence anonymity could not have been assured if staff had been categorised by education.

### Data collection

The first author distributed the questionnaires to the staff personally. All staff who were working in their ward during the three to four days of field observations were offered a questionnaire. Staff who worked night shifts only were excluded from the study because their work setting differed substantially from those of their colleagues; and staff who had worked less than eight weeks in their ward were excluded for lack of experience. Each staff member was offered a token gift (approximate value = 2 USD) along with the questionnaire. The questionnaires were completed anonymously and returned in sealed envelopes in a box located in the wards' staff room. A total of 444 questionnaires were returned, with a range of 5 to 19 respondents per ward and a mean of 11.4. The response rate from the 40 wards varied from 71% to 100%, with a total response rate of 87%.

Relatives answered a survey by phone interview. This survey was also conducted by the first author. Thirty five relatives were excluded due to limited contact with the resident or a complicated relationship between the relative and the resident. A total of 378 relatives agreed in to answer the questions, giving a response rate on 71%.

The first author's interviews with the 40 ward managers and 13 directors were performed in their offices in the course of the week of field observations. The interviews consisted of semi-structured questions. After the interviews, the ward managers answered a questionnaire consisting of specific questions about the ward.

The first author, with six years experience in nursing homes as an unlicensed worker, also conducted the field observations. Each ward was visited and observed for a total of 20 to 30 hours (within three to four days), depending on its size. A uniform was worn during the visits, and the author participated in the daily activities along with the staff. Both day and evening shifts were observed. During the field observations, notes were taken continuously on a PDA (Pocket PC), and the ward was scored according to predefined categories, as described below under the heading "Study variables". To avoid possible bias by a change in staff behaviour during the observations, anonymity was guaranteed to all staff participating in the study. The staff were also informed that the quality of care results would not be made available for the leaders of the nursing homes. A study by Schnelle et al. [55], indicates that staff behaviour is not influenced significantly by field observations.

### Study variables

Quality of care in Norway is regulated by *The national regulation for quality of care in nursing homes and home care *[23]. The regulation has been the starting point for indicators on quality of care in several studies [32-34]. According to the regulation, quality of care is a multidimensional phenomenon, consisting of a variety of aspects. Based on the regulation we developed four indicators: medical care, general care, social activities within the ward and social interactions between staff and residents. In addition we included a general indicator assessing the overall perception of the quality level - "all in all, how do you assess the quality of care at this nursing home ward" -, yielding a total of five quality of care indicators (see Table 1 for details). A general indicator has been used in several other composite scales in the health sector, like SF-36 [56]. The indicators were solely process and outcome measures [27], with an emphasis on outcome measures. Each indicator was measured by one to five items (see Appendix for details). Staff assessed nine items, relatives eight items and the field observer seven items. All items were measured on a scale ranging from one to seven, with 1 anchored at *strongly disagree *and 7 anchored at *strongly agree*.

**Table 1:** Descriptive statistics quality of care, scale 1 - 7 (N = 40 wards)

Relatives (N = 378 in 40 wards) (Cronbach's alpha = 0.92)	Mean	SD	Low - high
Medical care	5.27	0.65	3.40 - 6.50
General care	5.72	0.57	4.70 - 6.60
Social activities	3.92	1.07	1.60 - 5.75
Social interactions	5.37	0.74	4.09 - 6.50
General perception of quality of care	5.35	0.79	3.70 - 6.75
**Summary index**	**5.13**	**0.68**	**3.61 - 6.27**
**Staff **(N = 444 in 40 wards) (Cronbach's alpha = 0.85)			
Medical care	5.80	0.59	4.20 - 6.77
General care	5.32	0.52	4.03 - 6.44
Social activities	4.22	0.93	1.40 - 5.50
Social interactions	4.86	0.81	3.09 - 6.43
General perception of quality of care	5.49	0.74	4.00 - 6.86
**Summary index**	**5.14**	**0.58**	**4.03 - 6.15**
**Field observations **(N = 40) (Cronbach's alpha = 0.92)			
General care	5.65	0.61	4.00 - 7.00
Social activities	4.18	0.87	2.50 - 6.00
Social interactions	4.71	0.78	3.00 - 6.50
General perception of quality of care	5.00	0.82	3.00 - 6.00
**Summary index**	**4.89**	**0.69**	**3.38 - 6.25**

The responses from relatives, staff and the field observer formed three separate composite indexes (see Table 1 for details). The indexes were created by adding the indicators and calculating the mean value. The indexes based on the responses of relatives and staff contained all five quality indicators, while the index based on field observations did not include *medical care*, as the field observer has no medical education and since the field observations alone did not the put the field observer in a position to assess the medical care satisfactorily. Internal consistency of the indexes was high with Cronbach's alpha of 0.92 for relatives, 0.85 for staff and 0.92 for field observations, and supported the use of summary indexes. Factor analysis (we rotated the components using the Varimax method) showed that the three sources measured the quality of care significantly different - in accordance with prior studies of proxies and quality of care in nursing homes [34,57,58] (see Appendix for details).

*Leadership style *was measured by a scale based on selected items from Yukl [59], Northouse [43] and Bass & Stogdill [60], and was adapted to a nursing home setting. The instrument measured staffs' perceptions of their leaders' task- and relationship-oriented behaviours. The two leadership styles were each measured by five items on a scale ranging from 1 to 7, with 1 anchored at *strongly disagree *and 7 anchored at *strongly agree *(see Table 2). The individual data from each employee were aggregated to a ward level, creating a total of 40 different leadership styles. A factor analysis confirmed two leadership dimensions, namely task-oriented leadership style and relationship-oriented leadership style (see Appendix). The factor analysis and a strong support for a distinction between the two leadership styles in prior studies [35,37,40], support the use of two separate leadership dimensions. The internal consistency was high for both task-oriented leadership style (Cronbach's alpha 0.89) and relationship-oriented leadership (Cronbach's alpha 0.95).

**Table 2:** Descriptive statistics of the independent variables, scale 1 - 7 (N = 40 wards)

Leadership style (N = 444 staff in 40 wards)			
Task-oriented leadership style - Cronbach's alpha = 0.89	**Mean**	**SD**	**Low - high**
There are strict routines for refilling face cloth. towels and incontinence products	5.89	0.67	3.80 - 6.90
Reports during changing of personnel is preformed in a proper fashion	5.45	0.64	4.00 - 6.67
All personnel in the ward are aware of each individuals field of responsibility	5.11	0.83	1.80 - 6.60
The supervisor is concerned about the performance of routines. procedures and care plans	5.09	0.89	2.00 - 7.00
The supervisor is monitoring the performance of the tasks to make sure they are done properly	4.64	0.95	1.80 - 6.44
Relationship-oriented leadership style (scale 1 to 7) - Cronbach's alpha = 0.95			
The supervisor gives supports and encourages the staff	4.97	1.12	2.88 - 6.88
The supervisor is thoughtful towards the staff	5.06	1.17	2.38 - 6.86
The supervisor will be there for the staff if necessary	4.97	1.26	2.20 - 6.88
The supervisor listen to the staff	5.04	1.17	2.50 - 6.79
The supervisor is active in order to solve personal conflicts in the ward	4.75	1.18	1.20 - 6.45
**Staffing**			
Total staffing levels - number full time equivalents per residents per year (FTE)	0.81	0.08	0.67 - 0.99
Ratio of registered nurses	0.27	0.10	0.11 - 0.55
Ratio of unlicensed staff	0.19	0.10	0.05 - 0.50
**Care level**			
Percentage of residents dependent on wheel chair*	4.73	1.96	1 - 7
Percentage of residents dependent patient lift during care**	4.43	1.92	1 - 7

* = (1 = < 5%. 2 = 5% to 10%. 3 = 10% to 20%. 4 = 20% to 30%. 5 = 30% to 40%. 6 = 40% to 50% and 7 > 50%)

** = (1 = < 5%. 2 = 5% to 10%. 3 = 10% to 15%. 4 = 15% to 20%. 5 = 20% to 25%. 6 = 25% to 30% and 7 > 30%)

*The ratio of registered nurses *was measured by dividing the number of full time equivalents of registered nurses (FTE) in permanent positions in the ward by the total number of care workers (including unfilled posts). Only registered nurses directly involved with patient care were included. Consequently, ward managers and other registered nurses working within the administration were left out.

*The ratio of unlicensed staff *was measured by registering the actual number of unlicensed staff present at the ward during an average working day. The registering was done through a questionnaire filled in by the ward managers. This method was used due to the high number of vacant positions in nursing homes. Unlicensed staff are overrepresented in vacant positions [61] and the ratio of unlicensed staff would have been underestimated if we had based the measurement on the ratio of unlicensed staff in permanent positions.

*Total staffing levels *was measured by dividing the total full-time equivalent of care staff by the number of residents in the ward.

*Care level *was measured by two factors: *the percentage of residents dependent on wheel chair *and *the percentage of residents dependent on patient lift during care*, each of which was allocated a score from 1 to 7 (see Appendix for details). The data were obtained through field observations and interviews with care staff.

### Data analysis

We examined the level of collinearity among the independent variables and multicollinearity using the variance inflation factor (VIF) test. The correlations between the independent variables were low to moderate (*r <*0.50), except between task-oriented and relationship-oriented leadership styles (*r *= 0.78) (see Table 3). We did separate analyses for each of the three quality of care indexes. As the 40 wards were the units of the analyses, all data measured by staff and relatives were aggregated to ward level.

**Table 3:** Correlations (N = 40 wards)

	QoC - relatives	QoC - staff	**QoC - field obser**.	**Task-oriented leaders**.	**Relationship-oriented leaders**.	Total staffing levels	Ratio of registered nurses	Ratio of unlicensed staff	Care level
QoC - relatives	1								
QoC - staff	0.63**	1							
QoC - field observations	0.63**	0.65**	1						
Task-oriented leaders.	0.57**	0.71**	0.52**	1					
Relationship-oriented leaders.	0.50**	0.50**	0.45**	0.78**	1				
Total staffing levels	0.12	0.09	0.25	-0.02	0.00	1			
Ratio of registered nurses	0.08	-0.05	0.06	0.09	0.17	-0.14	1		
Ratio of unlicensed staff	-0.40*	-0.31	-0.50**	-0.32*	-0.37*	-0.09	-0.30	1	
Care level	-0.36*	-0.25	-0.22	0.13	0.22	-0.40*	0.26	-0.20	1

* (*p *< 0.05)

** (*p *< 0.01)

From earlier studies in nursing homes we know that organizational characteristics like ownership status, size and care level have effect on the level of quality of care [4,32,50,62,63]. As all nursing homes included in the present study were nonprofit and both owned and run by the local municipality, the ownership variable was irrelevant. Size and care level are both relevant variables, but due to our limited sample (N = 40) we choose to include only one of them. As size was less correlated with the three quality indexes (*r *= 0.28 versus *r *= 0.40), we included care level as a confounder. We additionally controlled for possible interaction effects among the independent variables [50,64]. The interaction effects were tested separately to limit the degrees of freedom. One significant interaction effect was found between task- and relationship-oriented leadership. The interaction effect was significant in the model with quality of care assessed by relatives only and is therefore not reported.

To account for clustering effects - as the 40 nursing home wards were located in 21 different nursing homes - we performed two-level analyses (random intercept models) [65]. A random intercept model allows the level of quality of care to vary across nursing homes and is suitable if there is nursing home-level variance that should be considered. The proportional reduction in variance (Pseudo R^2^) was assessed for ward and nursing home levels separately, as described by Snijders & Bosker [65].

Data were analyzed using SPSS (Statistical Program for Social Science) version 16 and mixed models were used for the analyses. For all statistical tests a 5% significance level was employed.

### Ethical considerations

The study has been approved by the Norwegian Social Science Data Services (NSD), an institution that assists and approves researchers with data gathering, data analysis, privacy issues and research ethics. All data in the study were anonymous, participation was voluntary and no separate data about any residents were collected. Participants were informed that confidentiality was assured and that they had the right to withdraw from the study at any point. Prior to field observations at the nursing homes, the first author made a declaration of nondisclosure of confidential information.

## Results

Table 1 presents the quality of care indexes and Table 2 presents descriptive statistics of the predictors used in the analyses. Concerning the assessments of the quality of care, one thing to note is that relatives and staff generally had a tendency to assess the quality to be higher than the field observer. A correlation matrix of the study variables is presented in Table 3 while the multilevel analyses testing the relationships between the quality indexes and the leadership and staffing variables are presented in Table 4.

**Table 4:** Two-level analyses (N = 40 wards and 21 nursing homes)

	Quality of care - relatives		Quality of care - staff		Quality of care - field observations	
	Coeff.	p-value	Coeff.	p-value	Coeff.	p-value
**Leadership style**						
Task-oriented leadership style	0.36	= 0.02	0.63	> 0.01	0.28	= 0.12
Relationship-oriented leadership style	0.12	= 0.19	0.01	= 0.91	0.10	= 0.37
						
**Staffing**						
Total staffing levels (FTE)	-0.95	= 0.31	0.10	= 0.90	1.17	= 0.30
Ratio of registered nurses	0.32	= 0.66	0.52	= 0.42	0.20	= 0.83
Ratio of unlicensed staff	-2.05	> 0.01	-0.80	= 0.22	-2.59	> 0.01
**Care level**	-0.20	> 0.01	-0.11	> 0.01	-0.11	= 0.02
R_1_^2 ^(ward level)	0.64		0.64		0.53	

The intra-class correlation coefficients (ICC) were 57%, 20% and 64% in the random intercept models with quality assessed by relatives, staff and field observations respectively. This shows that 57%, 20% and 64% of the total variability in quality of care was at the nursing home level and 43%, 80% and 36% at the ward level. These levels of ICCs' are large [65], and support the appropriateness of multilevel analyses.

The two-level analyses show that task-oriented leadership style was significantly related to quality of care as assessed by relatives (*p *= 0.02) and staff (*p *= < 0.01), but not significantly related to quality as assessed by field observations (*p *= 0.12). The lack of a significant relationship between task-oriented leadership style and the latter quality index is due to the strong correlation between task- and relationship-oriented leadership style (*r *= 0.78). In separate analyses of task-oriented leadership style and staffing data - conducted to test the isolated effect of the leadership style - task-orientation was significantly related to all three quality indexes (*p *< 0.01). Task-oriented leadership style showed the highest coefficient value in the model with quality assessed by staff and the lowest coefficient value in the model with quality assessed by field observations. Relationship-oriented leadership style was not significantly related to any of the quality indexes (*p *= 0.19, *p *= 0.91, *p *= 0.37). However, as for task-oriented leadership, separate analyses of relationship-oriented leadership showed a significant effect for relationship-oriented leadership style in all the three models (*p *< 0.01) and additionally, the bivariate correlations between relationship-oriented leadership style and the three quality indexes were strong and significant (*r *= 0.50, *r *= 0.50, *r *= 0.45). Consequently, the effect of relationship-oriented leadership in the multivariate analyses was erased by the strong correlation between the two leadership styles.

Total staffing levels and ratio of registered nurses were not significantly related to any of the quality indexes. Ratio of unlicensed staff was, however, significantly negatively related to quality as assessed by relatives and by field observations (*p *< 0.01), but not significantly related to quality as assessed by staff (*p *= 0.22). It is noteworthy that the ratio of unlicensed staff was the only predictor that was significantly differently related to the three quality indexes - all other predictors had a similar relationship across the indexes.

Care level showed a strong and significant negative relationship to all quality indexes (*p *< 0.01, *p *< 0.01, *p *= 0.02). The relationship was particularly strong for quality assessed by relatives.

The explanatory variables contributed to a 64% proportional reduction in variance between wards in the models where quality was assessed by relatives and staff and to 53% proportional reduction in variance between the wards in the model where quality was assessed by field observations.

## Discussion

The consistency of significant predictors in the present study was unexpected. Only one predictor - unlicensed staff - had a significant different effect across the three quality of care indexes. This consistency strengthens the predictors' validity and substantiates that although the factor analysis showed that relatives, staff and the field observer assessed the quality of care differently, these differences were not decisive with regard to their relationships to the predictors. The relatively high explained variance at the ward level (R_1_^2^) was a further surprise, as compared to earlier studies. An explained ward level variance of 64%, 64% and 53% in the models with quality assessed by relatives, staff and field observations respectively, shows that the predictors in the model explained much of the variation in quality of care between the wards and that common method variance was a minor problem in the study.

The strong correlation between task- and relationship-oriented leadership style (*r *= 0.78) - leaders who got high scores on task-oriented leadership also got high scores on relationship-oriented leadership and vice versa - showed that the two styles were closely related. This indicates that leadership style in the present study was to a certain extend a general phenomenon and that staff did not strongly differentiate between the two styles. However, factor analysis confirmed two separate leadership styles, showing that there was a significant distinction between the two styles. This distinction between leaders who focus on tasks and relationships, respectively, is in line with prior studies, both in the health sector [47] and in other organizations [35,37,40].

Relationship-oriented leadership style showed no significant effect on quality of care in the simultaneous analyses of the two leadership styles. This could indicate that relationship-oriented leadership style had no effect in the present study. However, this is not correct. Relationship-oriented leadership style had a significant effect on quality of care in separate analyses (*p *< 0.01), where the effect of task-oriented leadership was excluded. Consequently, the effect of relationship-oriented leadership style was erased by its strong correlation with task-oriented leadership style. Hence, we can conclude that both styles had a positive effect on quality of care in the present study and that the effect of task-oriented leadership style was the strongest.

The stronger effect of task-oriented leadership style was expected based on prior studies [35,37,40,42]. There might be unique contingencies in nursing homes though, that particularly facilitate the influence of task-oriented leadership style. In this regard we will propose two factors that are relatively constant for staff in nursing homes: 1) task-oriented leadership style has shown to be favourable for work which is characterized by high task structure and work that requires strong interdependency among staff [40,66,67]. Work in nursing homes has a typical task structure with defined and repetitive tasks and strong interdependency among staff is crucial for accomplishing daily tasks [8,68]. 2) Workplaces with shift work and activities 24/7 often have irregular workgroups and additionally the leader is normally present during day shift only. Such work settings may require more structure, planning and clarifying of roles than workplaces without shift work [69].

Concerning the effect of the two leadership styles on the different quality indexes, the particularly strong effect of task-oriented leadership style on staff assessed quality of care is worth mentioning. The findings are supported by Sellgren et al. [70], who showed that structure and clarity of roles - typical elements in task-oriented leadership style - were the most important leadership behaviour when staff assessed the quality of care.

The lack of any significant positive effect for staffing levels or ratio of registered nurses on the quality of care indexes stands in contrast to most prior studies [10,11]. An explanation for the present result may be the relatively high staffing levels and the relatively high ratio of registered nurses in Norwegian nursing homes compared with many other countries. There are approximately 0.80 full-time equivalents (FTE) of care workers per resident in Norwegian nursing homes and approximately 26% of them are registered nurses [33,61,71]. Studies from US and other Scandinavian countries report a considerably lower level of both staffing levels and ratio of registered nurses [52,64,72-74]. Thus, one plausible interpretation may be that the effects of increased staffing levels and increased ratio of registered nurses on quality of care are not linear at all staff levels (decreasing marginal productivity). The curve might flatten out, meaning that the positive effect of increased staffing levels and increased ratio of registered nurses on quality of care decreases above a certain level [13,75]. Zhang and Grabowski [76] and Abt Associates [77] found support for this assumption in a nursing home setting. Both studies have shown that the positive relationships between staffing and quality of care were significant in the lowest staffed nursing homes only.

Another explanation for the lack of effect could be the choice of quality measures used in the study. Arling et al. [2] suggested that *process *measures of quality may be more sensitive to staffing than *outcome *measures. This is because the outcome measures could be influenced by many other factors besides the defined predictors. In the literature review by Castle [11] 40% of the process indicators were found to be positively associated with staffing levels, as compared to 38% of the outcome measures. Yet, Castle [11] underscored that there was an extreme diversity in the quality indicators examined in the studies and that the type of quality measures may influence the effect of staffing. The majority of the quality of care items in the present study were *outcome *measures (see Appendix).

At last, it should be emphasized that the results concerning the effects of staffing levels and ratio of registered nurses on quality of care is ambiguous. Even if Castle [11] found a relationship between the two variables in most of the studies he examined in his literature review, 60% of the quality indicators in those studies were not related to increased staffing levels and 48% of the quality indicators were not related to increased level of registered nurses. Furthermore, the literature review by Spilsbury et al. [13] concluded that: "...research has produced inconsistent and contradictory results about the link between nurse staffing and quality in nursing homes." (p. 748).

In contrast to total staffing levels and ratio of registered nurses, the effect of unlicensed staff was significant; higher ratios of unlicensed staff had a negative effect on quality of care assessed by relatives and field observations. The effect of unlicensed staff on quality assessed by staff was not significant, however. The insignificant relationship (*p *= 0.22) may be explained by the assumption that licensed staff assessed the quality of care differently from unlicensed staff, the latter group presumably being less critical of their own work than licensed staff.

We should, however, be hesitant to conclude that unlicensed staff have a direct negative effect on quality of care. High ratios of unlicensed staff tend to be correlated with other factors that are unfavourable for quality of care, such as high staff turnover [50].

An increased physical care level showed a significant negative effect on all three quality indexes. The relative effect was strongest when quality was assessed by relatives. The strong effect of care level may have two potential explanations: 1) nursing homes provide better care to residents with high physical functional ability than to residents with low physical functional ability and/or 2) the physical functional ability level of the residents influence the assessments of quality of care, and in particular the assessments made by relatives.

The ICCs' of 57%, 20% and 64% showed that there was a substantial clustering of wards within nursing homes (see Table 4). Future research is encouraged to use a design that makes it possible to identify both nursing home and ward factors that influence quality of care. A factor of interest is the leadership behaviour of the director of the nursing home, [1] and a simultaneously assessment of the ward leaders and the director of the nursing homes leadership styles would have been of particular interest.

There are several limitations to our study. First, the number of participating nursing home wards is limited and our sample is not representative of the population of Norwegian nursing homes although regions and communities all over the country were included. Second, the quality of care was assessed by self-reported data only. A potential weakness with primary data sources is the subjective aspect - it depends upon the individuals who carry out the assessment. The inclusion of secondary data sources would have been beneficial for the study. However, Norway has no national data sets like the MDS and other secondary data sources were not available. To compensate for the lack of secondary data sources, we used three independent primary data sources; relatives, staff and field observations. It should be emphasized that these sources are well suited for measuring quality of care in nursing homes [13,21,22,34,78-81]. Third, the study had a cross-sectional design which does not take into account the relatively high turnover rate in the sector. Longitudinal design could have strengthened the study; though not possible within the scope of this investigation.

## Conclusion

This study indicates that the relationship between staffing levels, ratio of registered nurses and quality of care may be more complex than some prior research suggests. Increasing staffing levels or the ratio of registered nurses above a certain level is not likely to be sufficient for raising quality of care. The study also shows that nursing homes should aim to minimize the use of unlicensed staff where possible by addressing the underlying reasons for the use of such staff, such as high staff turnover. The significant positive effect of leadership styles on quality of care underlines the importance of active leadership in nursing homes. The stronger effect for task-oriented leadership style suggests that leaders in nursing homes should in particular focus on task-oriented conditions like structure, coordination, clarifying of roles and monitoring of operations.

## Competing interests

The authors declare that they have no competing interests.

## Authors' contributions

AKH and TIR designed the study. AKH collected the data, performed the field observations, analysed the data and wrote the first draft of the paper. AS, TIR and LEK participated in the interpretation of the data analysis and critically commented the first draft. All authors approved the final manuscript.

## Appendix

See Tables 5, 6 and 7 in Appendix.

**Table 5:** Quality of care - details

Quality of care items (N = 40 wards)
**Medical care**
How satisfied are you with the medical care at the nursing home? *(relatives)*
The ward has relevant procedures in relation to death *(staff)*
The ward gives good care to terminal residents *(staff)*
There is a considerable professional medical focus at the ward *(staff)*
All pressures ulcer are treated adequately and promptly *(staff)*
**General care**
How satisfied are you with the daily care at the nursing home? *(relatives)*
How satisfied are you with the personal hygiene at the nursing home? *(relatives)*
Do the staff act with dignity and respect during care? *(relatives)*
The staff note all relevant care information in the journal *(staff)*
The resident get their teeth brushed every day *(staff)*
Respect for residents during care activities *(field observations)*
Social atmosphere between residents and staff during care *(field observations)*
**Social activities**
How satisfied are you with the level of activities offered to the residents? *(relatives)*
The residents are offered a sufficient level of daily activities *(staff)*
General level of activities for the residents *(field observations)*
The staff arrange coffee breaks etc *(field observations)*
**Social interactions**
How satisfied are you with the staff's behaviour towards the residents *(relatives)*
Do the staff carry conversations with the residents? *(relatives)*
Do the staff carry conversations with the residents *(staff)*
To what degree do the staff carry conversations with the residents? *(field observations)*
Empathy showed by the staff towards the residents *(field observations)*
**General perception**
All in all. how satisfied are you with the care at the nursing home *(relatives)*
All in all. how would you assess the care the residents receive at this nursing home *(staff)*
General assessment of the care at the nursing home *(field observations)*

**Table 6:** Factor analysis - relatives, staff and field observations

Factor analysis - Rotated Component Matrix (N = 40 wards)			
	Component		
	**1**	**2**	**3**
	
Medical care (relatives)	**0.853**		
General perception (relatives)	**0.828**	0.409	
Social interactions (relatives)	**0.799**	0.372	
Social activities (relatives)	**0.785**		
General care (relatives)	**0.778**	0.431	
Social interactions (field observations)		**0.871**	
General perception (field observations)		**0.861**	
Social activities (field observations)	0.384	**0.771**	
General care (field observations)		**0.738**	0.402
Social activities (staff)			**0.760**
General care (staff)			**0.747**
Social interactions (staff)	0.527		**0.667**
General perception (staff)	0.419	0.471	**0.631**
Medical care (staff)	0.448	0.439	**0.589**

**Table 7:** Factor analysis - leadership style

Factor analysis - Rotated Component Matrix (N = 444 staff)		
Task-oriented = TO, Relationship-oriented = RO	Component	
	
	1	2
The supervisor is thoughtful towards the staff (RO)	**0.909**	
The supervisor will be there for the staff if necessary (RO)	**0.895**	
The supervisor listen to the staff (RO)	**0.894**	
The supervisor gives supports and encourages the staff (RO)	**0.877**	
The supervisor is active in order to solve personal conflicts in the ward (RO)	**0.831**	
The supervisor is monitoring the performance of the tasks to make sure they are done properly (TO)	0.640	**0.542**
There are strict routines for refilling face cloth. towels and incontinence products (TO)		**0.770**
Reports during changing of personnel is preformed in a proper fashion (TO)		**0.740**
All personnel in the ward are aware of each individuals field of responsibility (TO)		**0.734**
The supervisor is concerned about the performance of routines. procedures and care plans (TO)	0.568	**0.623**

## Pre-publication history

The pre-publication history for this paper can be accessed here:

http://www.biomedcentral.com/1472-6963/11/327/prepub
